# Generative adversarial network-created brain SPECTs of cerebral ischemia are indistinguishable to scans from real patients

**DOI:** 10.1038/s41598-022-23325-3

**Published:** 2022-11-05

**Authors:** Rudolf A. Werner, Takahiro Higuchi, Naoko Nose, Fujio Toriumi, Yohji Matsusaka, Ichiei Kuji, Koshino Kazuhiro

**Affiliations:** 1 Department of Nuclear Medicine, University Hospital Würzburg, Oberdürrbacher Str. 6, 97080 Würzburg, Germany; 2 The Russell H Morgan Department of Radiology and Radiological Sciences, Division of Nuclear Medicine and Molecular Imaging, Johns Hopkins School of Medicine, Baltimore, MD USA; 3 Graduate School of Medicine, Dentistry and Pharmaceutical Sciences, Okayama University, Okayama, Japan; 4 Department of Systems Innovation, Graduate School of Engineering, The University of Tokyo, Bunkyo-Ku, Japan; 5 Department of Nuclear Medicine, Saitama Medical University International Medical Center, Saitama, Japan; 6 Department of Systems and Informatics, Hokkaido Information University, Ebetsu, Japan

**Keywords:** Machine learning, Computational biology and bioinformatics, Neurology

## Abstract

Deep convolutional generative adversarial networks (GAN) allow for creating images from existing databases. We applied a modified light-weight GAN (FastGAN) algorithm to cerebral blood flow SPECTs and aimed to evaluate whether this technology can generate created images close to real patients. Investigating three anatomical levels (cerebellum, CER; basal ganglia, BG; cortex, COR), 551 normal (248 CER, 174 BG, 129 COR) and 387 pathological brain SPECTs using N-isopropyl p-I-123-iodoamphetamine (^123^I-IMP) were included. For the latter scans, cerebral ischemic disease comprised 291 uni- (66 CER, 116 BG, 109 COR) and 96 bilateral defect patterns (44 BG, 52 COR). Our model was trained using a three-compartment anatomical input (dataset ‘A’; including CER, BG, and COR), while for dataset ‘B’, only one anatomical region (COR) was included. Quantitative analyses provided mean counts (MC) and left/right (LR) hemisphere ratios, which were then compared to quantification from real images. For MC, ‘B’ was significantly different for normal and bilateral defect patterns (*P* < 0.0001, respectively), but not for unilateral ischemia (*P* = 0.77). Comparable results were recorded for LR, as normal and ischemia scans were significantly different relative to images acquired from real patients (*P* ≤ 0.01, respectively). Images provided by ‘A’, however, revealed comparable quantitative results when compared to real images, including normal (*P* = 0.8) and pathological scans (unilateral, *P* = 0.99; bilateral, *P* = 0.68) for MC. For LR, only uni- (*P* = 0.03), but not normal or bilateral defect scans (*P* ≥ 0.08) reached significance relative to images of real patients. With a minimum of only three anatomical compartments serving as stimuli, created cerebral SPECTs are indistinguishable to images from real patients. The applied FastGAN algorithm may allow to provide sufficient scan numbers in various clinical scenarios, e.g., for “data-hungry” deep learning technologies or in the context of orphan diseases.

## Introduction

In recent years, the use of artificial intelligence (AI) based on neural networks in medical imaging has been rapidly expanding. Improved diagnostic accuracy of this technology is tightly linked to enlarged network models, which are also associated with higher costs, computing to train AI and increasing efforts of data annotation, e.g., by human experts^1,2^. As such, data augmentation based on processing techniques provides images similar to supervised data, e.g., by applying geometric deformation, brightness, saturation changes, random cropping, or mix-ins to natural images^3^. Of note, such conventional techniques of data expansion may be limited for molecular or conventional imaging, mainly caused by asymmetry of organ shapes and locations, less standardized protocols for patient’s orientation, constraints of quantifications, or varying receptor density, glucose consumption or blood flow on a subcellular level^3,4^.

Relative to the afore-mentioned methods of data augmentation applying direct alterations to existing images, generative adversarial networks (GANs) are based on artificial neural networks to create images closely following the feature distributions of a set of supervised images. In brief, those neural networks consist of generator and discriminator: a generator produces images with features resembling real-world images, and a discriminator segregates between real and the generated images^5^. GAN uses a simple training strategy of competing generator and discriminator against each other to synthesize images closely resembling real ones. In this regard, GAN is a promising technology for medical imaging, and has been actively studied for various purposes such as data augmentation, modality conversion, segmentation, super-resolution, denoising and reduction of radiation exposure for medical imaging^4,6–11^. Focusing on data augmentation, prior studies of GAN in magnetic resonance imaging have reported on the generation of 2D images with lesions^12^, generation of 3D images^13^, conversion of abnormal images to normal images^14^, and synthesis of brain images that reflect age-related changes^15^. In addition, previous studies on positron emission tomography images have also demonstrated that image generation by independently learning images of different stages of cognitive decline are feasible (including normal cases, mild cognitive impairment, and Alzheimer’s disease)^16^. Moreover, in the field of neuroimaging, previous investigators focused on conversion of ^11^C Pittsburgh compound B images^17^, or ^18^F-florbetapir^18^, e.g., to obtain sufficient number of training cases for computer-aided diagnosis. Taken together, in most of those studies, GAN-generated images were then applied to augment imbalanced datasets or “data-hungry” deep learning technologies, without the need of labeling by expert readers. Prior to a more widespread adoption of this technology, however, such generated scans should be validated, e.g., by comparing with real images, preferably among a broad spectrum of different disease conditions^19^. In this regard, the number of needed scans increases relative to the number of different conditions attributed to the underlying disease. For instance, in patients with cerebral ischemia using N-isopropyl p-I-123-iodoamphetamine (^123^I-IMP) SPECT, various defect patterns can be recorded, e.g., affecting only one hemisphere or global reduced blood flow^20^. Minimizing the number of training data fed into GAN, however, would be desirable, as it would enable for an increased use of this application even if only a small sample size of supervised images is available.

To address this issue, a light-weight GAN (FastGAN) has been recently proposed to enable learning with a smaller set of supervised real data, thereby allowing to reduce the number of initially provided items serving as stimuli^21^. Incorporating a conditioning mechanism into FastGAN, we aimed to generate brain images of uni- and bilateral cerebral ischemia using ^123^I-IMP SPECT. Created scans were then validated by quantitative comparison with images of real patients, which allowed to determine whether FastGAN-based scans of reduced cerebral blood flow resemble their real equivalents.

## Material and methods

### SPECT procedures and training data

250 patients (age, 61.0 ± 16.4 years, 96/250 (38.4%) female), which had undergone ^123^I-IMP brain SPECTs to assess cerebral ischemia were included in this retrospective analysis. Given the retrospective nature of this study, informed consent was waived by the institutional review board at Saitama Medical University International Medical Center (#2022-016), which also approved the study. All procedures were carried out following current guidelines^22^. Images were performed under rest and stress condition at one day and thus, a total of 500 scans were available for analyses. We used a Siemens Symbia 16 SPECT/CT system (Siemens Healthineers, Erlangen, Germany), equipped with the quantitative SPECT (QSPECT) reconstruction program and split-dose autoradiographic (ARG) method. SPECT scanning was performed using a low-energy high-resolution collimator with a SPECT condition of continuous rotation at 90 views, 2 min/rotation, 2 cycles of 7 repeats. Data were reconstructed according to the quantitative assessment of rest- and acetazolamide-CBF QSPECT/DTARG protocol^23,24^. Matrix size of the slices was 64 × 64 and each SPECT image was reoriented perpendicular to the anterior–posterior commissure line. Detailed image reconstruction is also provided in^25^. Slices at three anatomical levels including cerebellum (CER), basal ganglia (BG), and cortex (COR) were selected from whole brain images by a board-certified nuclear medicine physician (T.H.). The number of selected slices for defect patterns and slice levels are shown in Table 1. Two datasets were created. For dataset ‘A’, we used a three-compartment anatomical input, including CER, BG, and COR, while for dataset ‘B’, only one anatomical region (COR) was considered.

**Table 1:** Number of slices for patterns of radiotracer accumulation and slice levels in the training dataset.

	Radiotracer accumulation		
	Normal	Unilateral	Bilateral
**Anatomical level**			
Cerebellum	248	66	0
Basal ganglia	174	116	44
Cortex	129	109	52
Total	551	291	96

### Network model

For our network model, we applied the previously published FastGAN^21^ to conditional GAN^19^ with modification for specifying defect pattern and adaptation to image matrix size. In brief, the quality of the generated images was improved by alternately training a generator that synthesizes images and a discriminator assessing the authenticity of images.

### Generator

**Figure 1:**
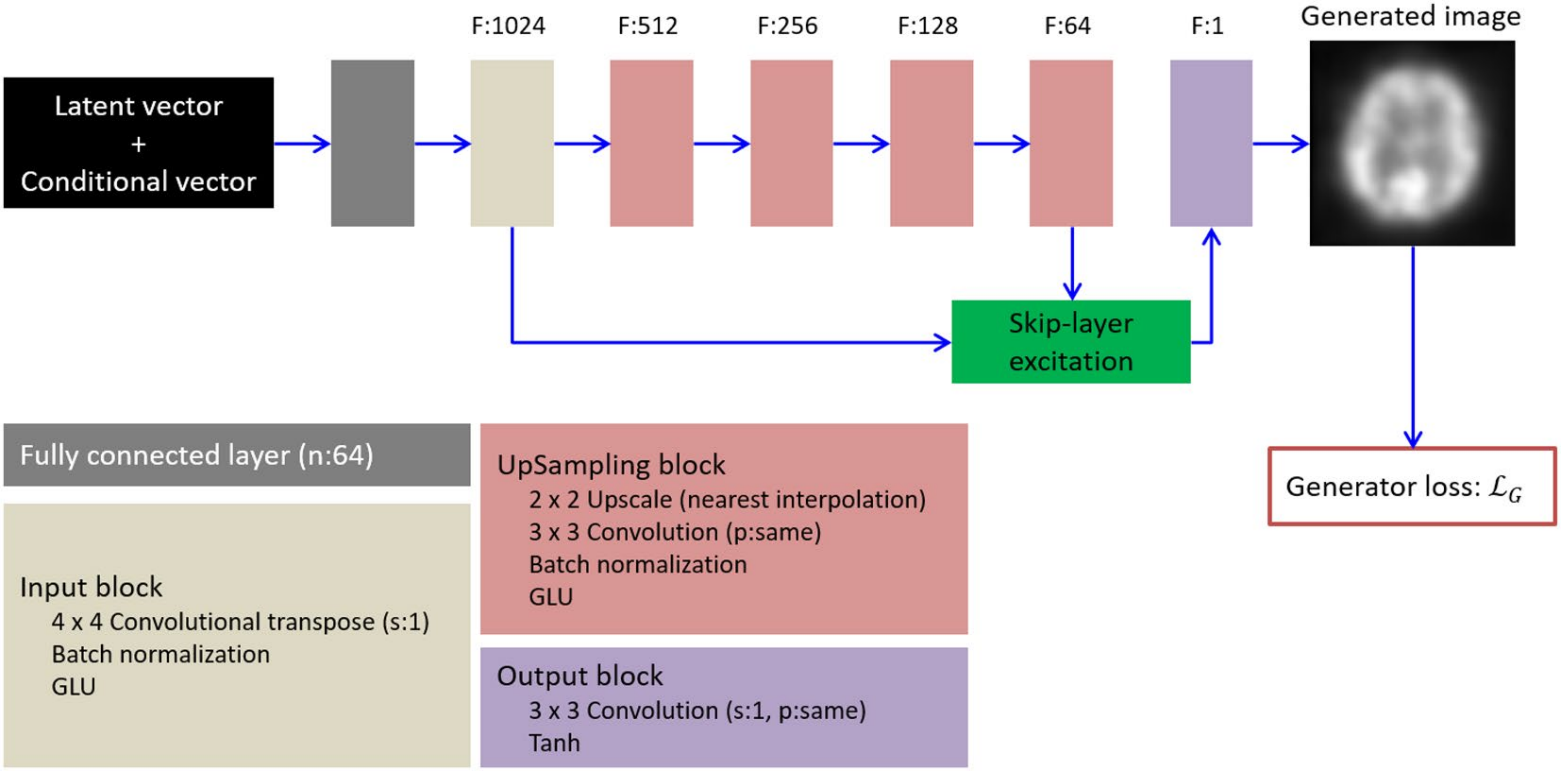
Generator network in our model. The latent vector and the conditional vector for specifying patterns of radiotracer accumulation served as input to the generator to synthesize a two-dimensional brain SPECT. Symbols F, n, s and p denote channels of output feature maps, number of neurons, strides and padding, respectively. The “same” for padding indicates that padding is applied to the input feature map so that the height and width of the input and output feature maps are not changed. GLU is a gating unit proposed in^42^. Tanh is a hyperbolic tangent activation function. Loss function ${\mathcal{L}}_{G}$ is defined as Eq. (1).

**Figure 2:**
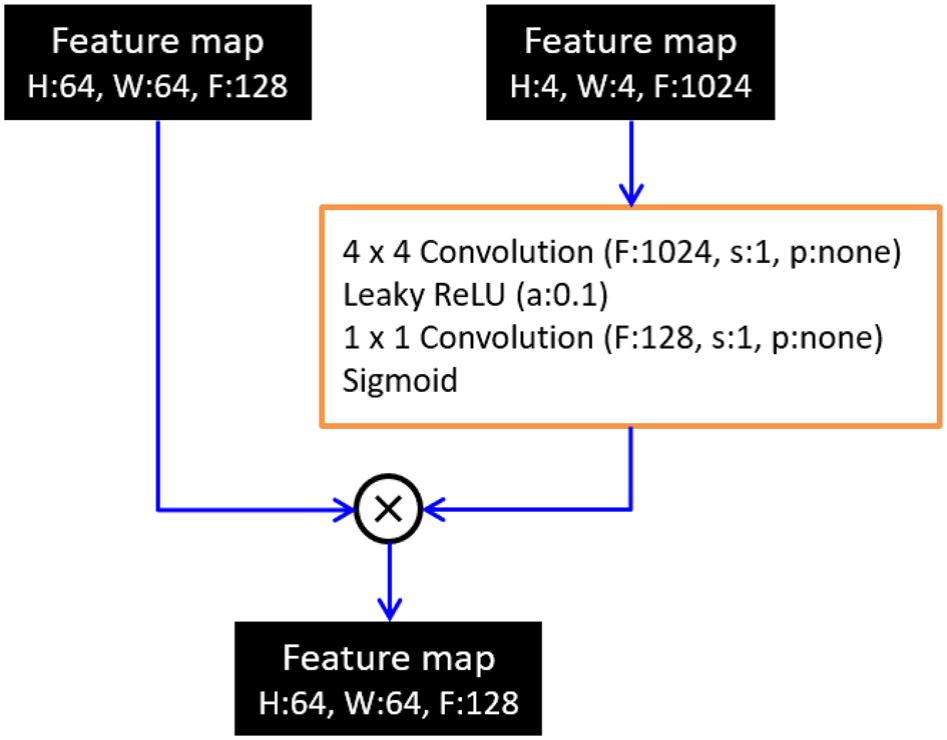
Skip-layer excitation module used in the generator. Symbols H, W and F in feature maps denote height, width and channels, respectively. Symbols s, p and a denote strides, padding and slope of Leaky ReLU activation function, respectively. For padding, “none” indicates that no padding is applied to the input feature map.

For the generator, latent and conditional vectors for specifying defect patterns were taken as input (Fig. 1). In this regard, the latent vector was used for ensuring variety of generated images (vector dimensions, 256). The conditional vector for the dataset ‘A’ included slice levels and cerebral patterns of radiotracer accumulation (as 1 or 0). In this regard, the first three elements corresponded to anatomical levels, while the patterns included normal, uni- and bilateral defects. The conditional vectors for the dataset ‘B’ included only the COR, along with the same defect patterns (Table 2). The generator consisted of blocks with four different roles and a skip-layer excitation module. The fully connected layer embedded the input vector into a 64 dimensional vector. This layer also adjusted the data dimension so that the input block is acceptable. Both the input and up-sampling blocks enlarged the feature maps progressively to produce more detailed image, while the output block generated a monochromatic brain SPECT image from the input feature maps. Moreover, the skip-layer excitation module enabled efficient training of the generator through gradient flow between distant layers and self-gating^21^. Different from the previously published FastGAN, our model used only one skip-layer excitation, as such an approach allowed for reducing the number of parameters needed to be learned along with a small matrix size of input images. Additionally, adaptive pooling layer is omitted as it is considered unnecessary for low-resolution images (Fig. 2).

**Table 2:** Conditional vectors for the dataset ‘A’ and ‘B’.

	Radiotracer accumulation		
	Normal	Unilateral	Bilateral
**Anatomical level**			
Dataset ‘A’			
Cerebellum	(1 0 0 1 0 0)	(1 0 0 0 1 0)	(1 0 0 0 0 1)
Basal ganglia	(0 1 0 1 0 0)	(0 1 0 0 1 0)	(0 1 0 0 0 1)
Cortex	(0 0 1 1 0 0)	(0 0 1 0 1 0)	(0 0 1 0 0 1)
Dataset ‘B’			
Cortex	(1 0 0)	(0 1 0)	(0 0 1)

Loss function of the generator ${\mathcal{L}}_{G}$ was given by^21^:

1 $${\mathcal{L}}_{G}=-{\mathbb{E}}_{z\sim \mathcal{N}}\left[D\left(G\left(z|y\right)\right)\right]$$

where $z$ was a latent vector sampled from standard normal distribution, $y$ was a conditional vector, $G\left(z|y\right)$ was a synthesized image by the generator and $D\left(\cdot \right)$ was real/fake logits for $G\left(z|y\right)$ predicted by the discriminator.

### Discriminator

**Figure 3:**
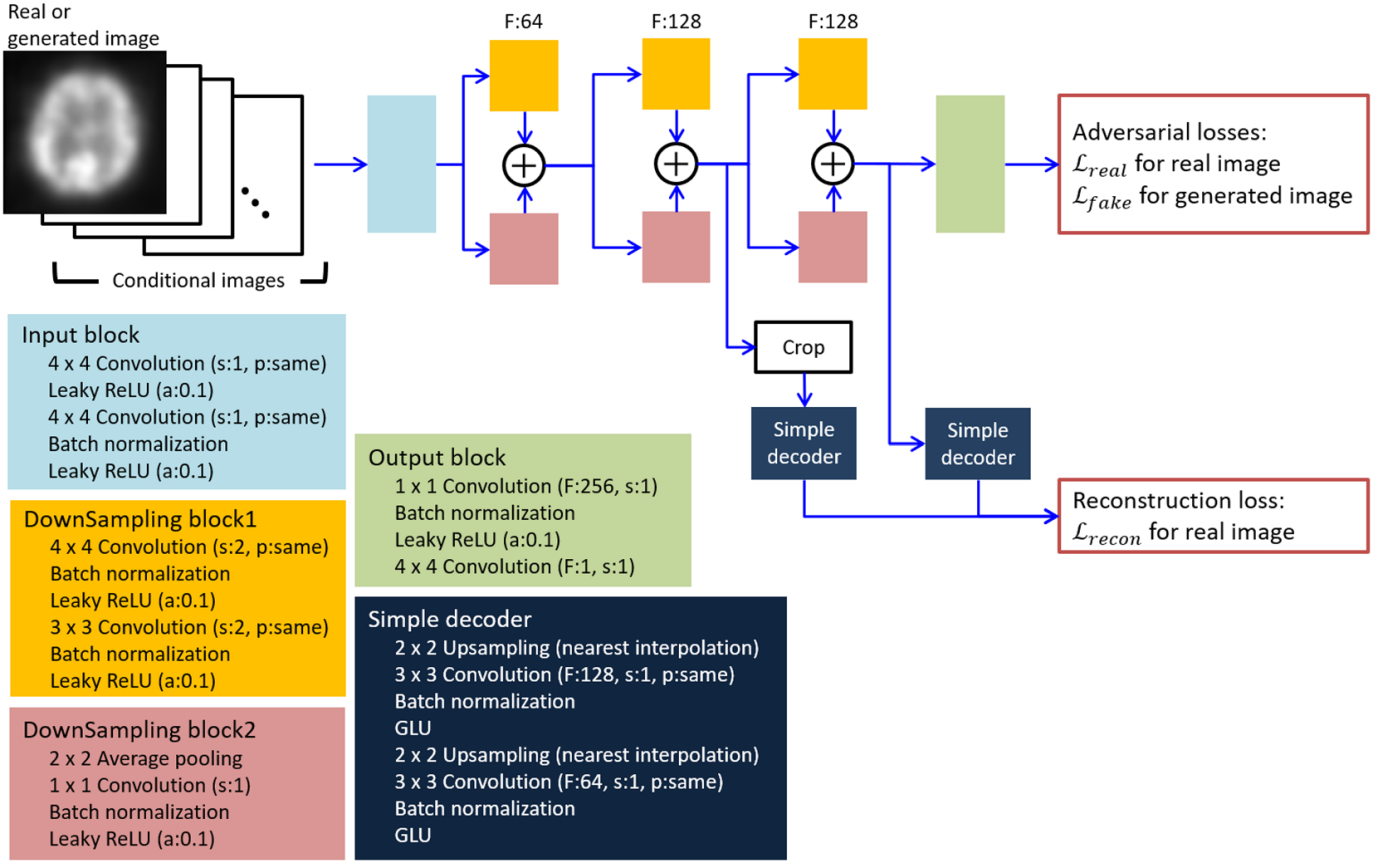
Discriminator in our model. The discriminator uses as input the real or generated image with the conditional image representing the pattern of radiotracer accumulation. Symbols F, s, p and a denote channels of output feature maps, strides, padding and slope of Leaky ReLU activation function, respectively. For padding, the “same” indicates that padding is applied to the input feature map so that the height and width of the input and output feature maps are not changed, “none” indicates that no padding is applied to the input feature map. GLU is a gating unit proposed in^42^. Loss function ${\mathcal{L}}_{real}$, ${\mathcal{L}}_{fake}$ and ${\mathcal{L}}_{reocn}$ are defined as Eqs. (2, 3, 4), respectively.

For the discriminator, two types of images (one being real and one being generated) served as inputs which had to be discriminated (Fig. 3). For specifying the pattern of radiotracer accumulation, a conditional image was applied, in which number of channels were 6 and 3 for the dataset ‘A’ and ‘B’, respectively. Each channel corresponded to an element of the conditional vector in the generator, and all pixel values in a channel are 0 or 1. The input image was processed through input and down-sampling blocks to extract the features of the real image, while the output block processed the feature maps from the last down-sampling block, thereby assessing the probability that the input image was real as the following Equations^21^.

2 $${\mathcal{L}}_{real}=-{\mathbb{E}}_{x\sim {I}_{real}}\left[\mathrm{min}\left(0,-1+D\left(x\right)\right)\right]$$

3 $${\mathcal{L}}_{fake}=-{\mathbb{E}}_{\widehat{x}\sim G\left(z|y\right)}\left[\mathrm{min}\left(0,-1-D\left(\widehat{x}\right)\right)\right]$$

where ${\mathcal{L}}_{real}$ and ${\mathcal{L}}_{fake}$ were adversarial loss for real and generated images. $x$ and $\widehat{x}$ were sampled from real images ${I}_{real}$ and the generated images $G\left(z|y\right)$, respectively. $D\left(x\right)$ represented real/fake logits for the input $x$.

To efficiently learn features of real images, self-supervised learning was employed with cropping and simple decoders. Briefly, regional feature maps with half height and half width were cropped at a random location of the feature map from the second down-sampling block. The feature map and the global feature map from the third down-sampling block were input to the simple decoders to reconstruct the regional and whole real image from these feature maps. The similarities between the reconstructed image and a real image at regional and global levels and the same location in the real image were evaluated by^21^:

4 $${\mathcal{L}}_{recon}={\mathbb{E}}_{x\sim {I}_{real}}\left[\Vert {\mathcal{G}}_{1}\left({\mathcal{B}}_{1}\left(x\right)\right)-\mathcal{T}\left(x\right)\Vert +\Vert {\mathcal{G}}_{2}\left({\mathcal{B}}_{2}\left(x\right)\right)-x\Vert \right]$$

The loss ${\mathcal{L}}_{recon}$ was evaluated on only real images. ${\mathcal{B}}_{1}\left(x\right)$ and ${\mathcal{B}}_{2}\left(x\right)$ was feature maps from the second and third down-sampling block, ${\mathcal{G}}_{1}\left(\cdot \right)$ was a function contained cropping and processing by the decoder on ${\mathcal{B}}_{1}\left(x\right)$, $\mathcal{T}\left(x\right)$ was a function of cropping on sample $x$, and ${\mathcal{G}}_{2}\left(\cdot \right)$ was a function by the decoder on ${\mathcal{B}}_{2}\left(x\right)$.

Total loss of the discriminator ${\mathcal{L}}_{D}$ was given by:

5 $${\mathcal{L}}_{D}={\mathcal{L}}_{real}+{\mathcal{L}}_{fake}+{\mathcal{L}}_{recon}$$

### Training process

Each slice was normalized by the maximum count of the slice. To increase number of samples, the following data augmentation was performed: for weighted averaged slices, $z$ was calculated using the target slice ${z}_{1}$ and a slice ${z}_{2}$,

6 $$z=w\times {z}_{1} + (1-w) \times {z}_{2}$$

where ${z}_{2}$ was randomly selected from adjacent in craniocaudal direction, while weight $w$ was a random number ranging from 0 to 1. The weighted average slice was translated in anteroposterior direction by $t$ pixel. The value of $t$ was a random integer from -2 to 2. A horizontal flip was applied to real slices of normal and bilateral patterns as the discriminator inputs at random. The generator and discriminator were trained alternately in the following steps: (i) Synthesized images were outputted by the generator. (ii) The loss of the discriminator to the real image was calculated based on Eqs. (3,5). (iii) The loss of the discriminator to the generated image was calculated based on Eq. (4). (iv) The total loss of the discriminator was computed based on Eq. (2). (v) The loss of the generator was calculated based on Eq. (1). (vi) The parameters of the generators and discriminators were updated using the corresponding losses and Adam optimizers^26^ with ${\upbeta }_{1}=0.9$, ${\upbeta }_{2}=0.999$ and learning rates = $2\times {10}^{-4}$. Our model was trained with 1000 epochs and batch size 4 using the dataset ‘A’ and ‘B’, independently on a single NVIDIA RTX 2080 GPU and TensorFlow 2.2.

### Testing of both datasets for resembling real images

The epochs, where highest accuracy for real images were given, were determined by a board-certified nuclear medicine physician with 10 years of experience (T.H.) for the two trained models. Using the model parameters at the determined epochs, the same number of images as the real ones were generated for normal, uni- and bilateral conditions as described in Table 1. To evaluate the fidelity of the generated images by our GAN with parameters of the selected epoch, we calculated mean counts (MC), average of pixel-wise standard deviation (SD), and count ratios of left to right hemisphere (LR) for real and generated images trained with datasets ‘A’ and ‘B’. Inter-slice variation within each pattern of real and generated images was assessed using pixelwise SD map.

### Statistical analysis

For comparing real images and images of both datasets ‘A’ and ‘B’, we applied one-way ANOVA with GraphPad Prism 9 (San Diego, CA, USA). A *P* < 0.05 was considered statistically significant.

## Results

**Figure 4:**
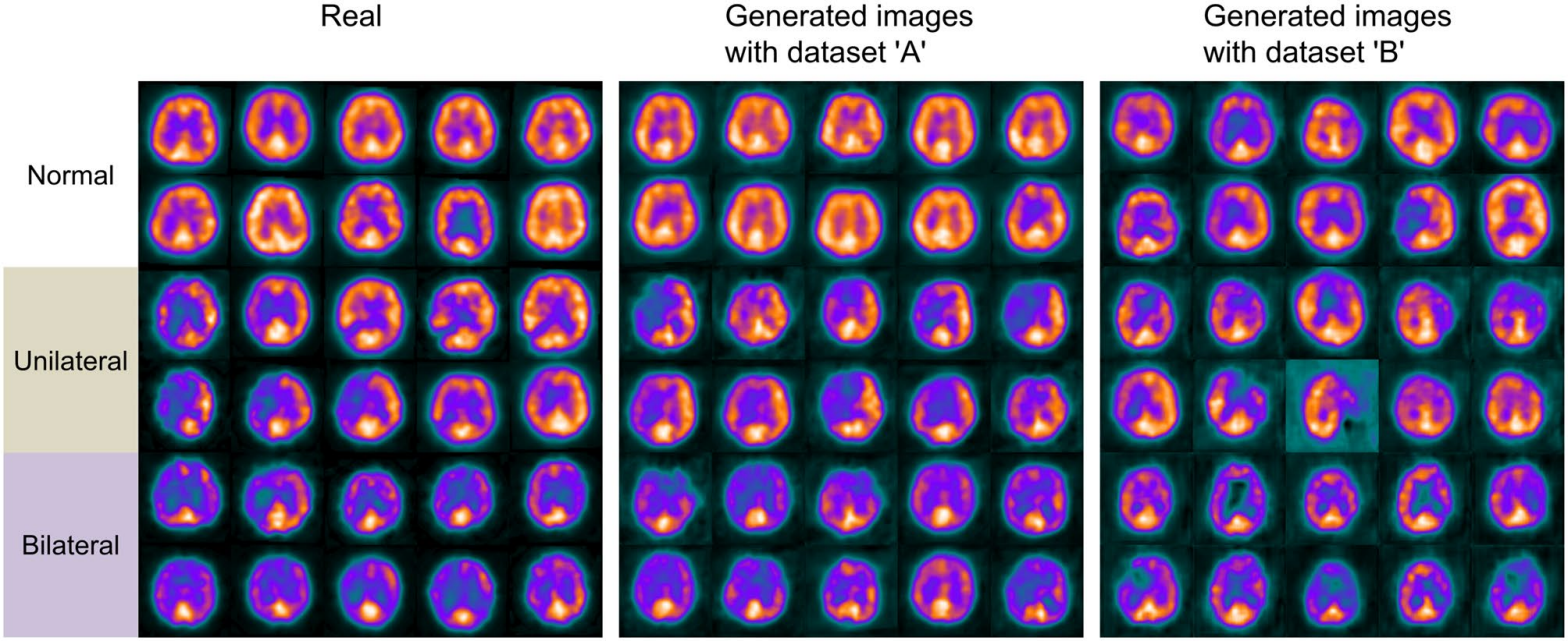
Real images, generated images trained with dataset ‘A’ (with three-compartment levels serving as stimuli), and dataset ‘B’ (only providing one anatomical level as input). On a visual assessment, dataset ‘A’ including more anatomical information resembles real images more closely than generated images by dataset ‘B’.

Epochs with highest accuracy were 826 and 651 for dataset ‘A’ and ‘B’, respectively. On a visual assessment, image quality of generated images with dataset ‘A’ was superior when compared to ‘B’ (Fig. 4).

**Figure 5:**
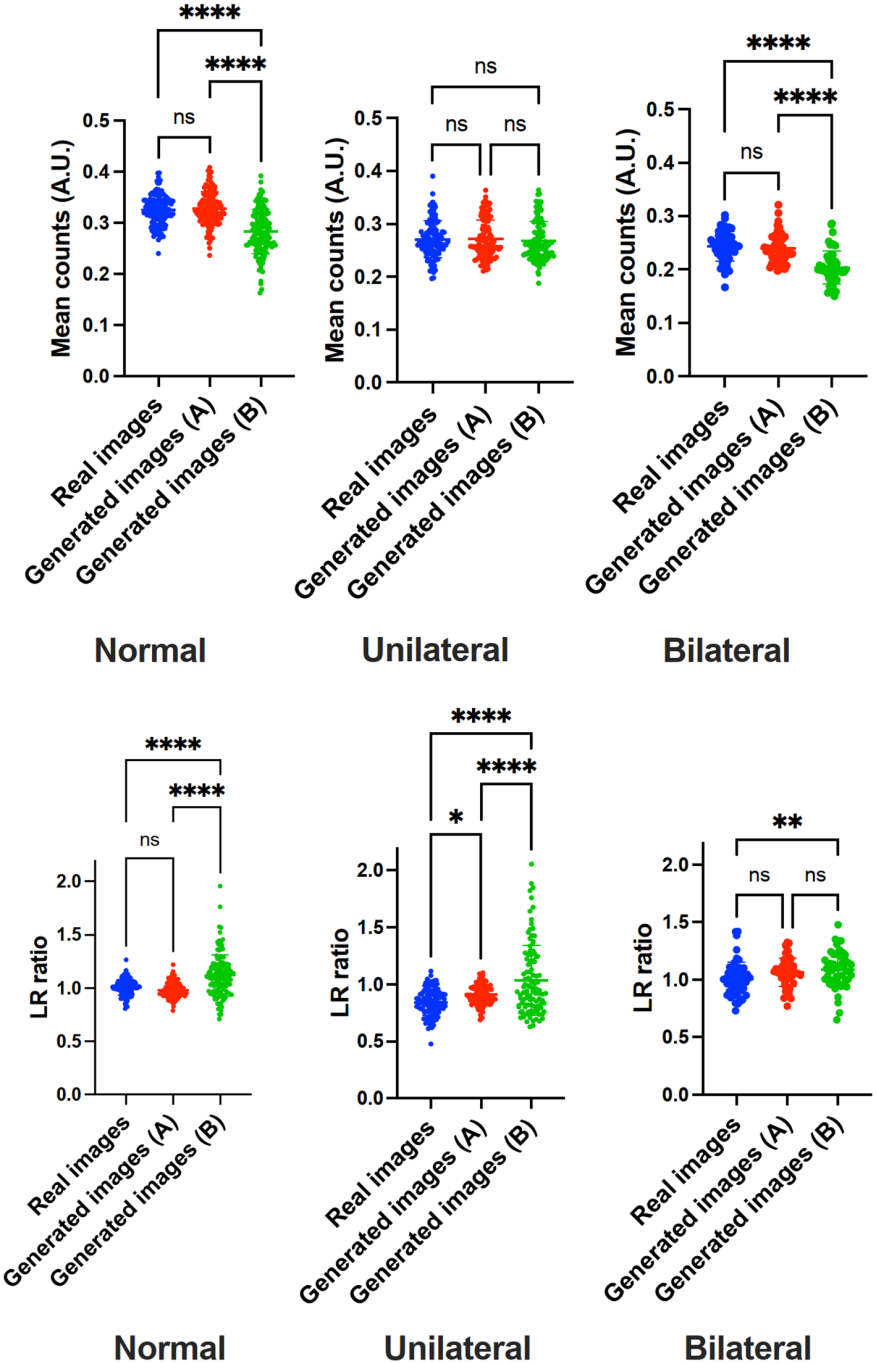
Whisker Plots for comparing real images and generated images for datasets ‘A’ and ‘B’. First row: mean counts, second row: left to right hemisphere ratio (LR). Except for unilateral defect patterns on LR, all comparisons of ‘A’ with real images failed to reach significance. On the other hand, for dataset ‘B’, statistical significance was reached in almost all cases (expect for mean counts of unilateral ischemia), supporting the notion that ‘A’ (using more anatomical input) provides scans closely resembling real scans. *, ** and **** denote *P* < 0.05, *P* < 0.01 and *P* < 0.0001, respectively.

**Figure 6:**
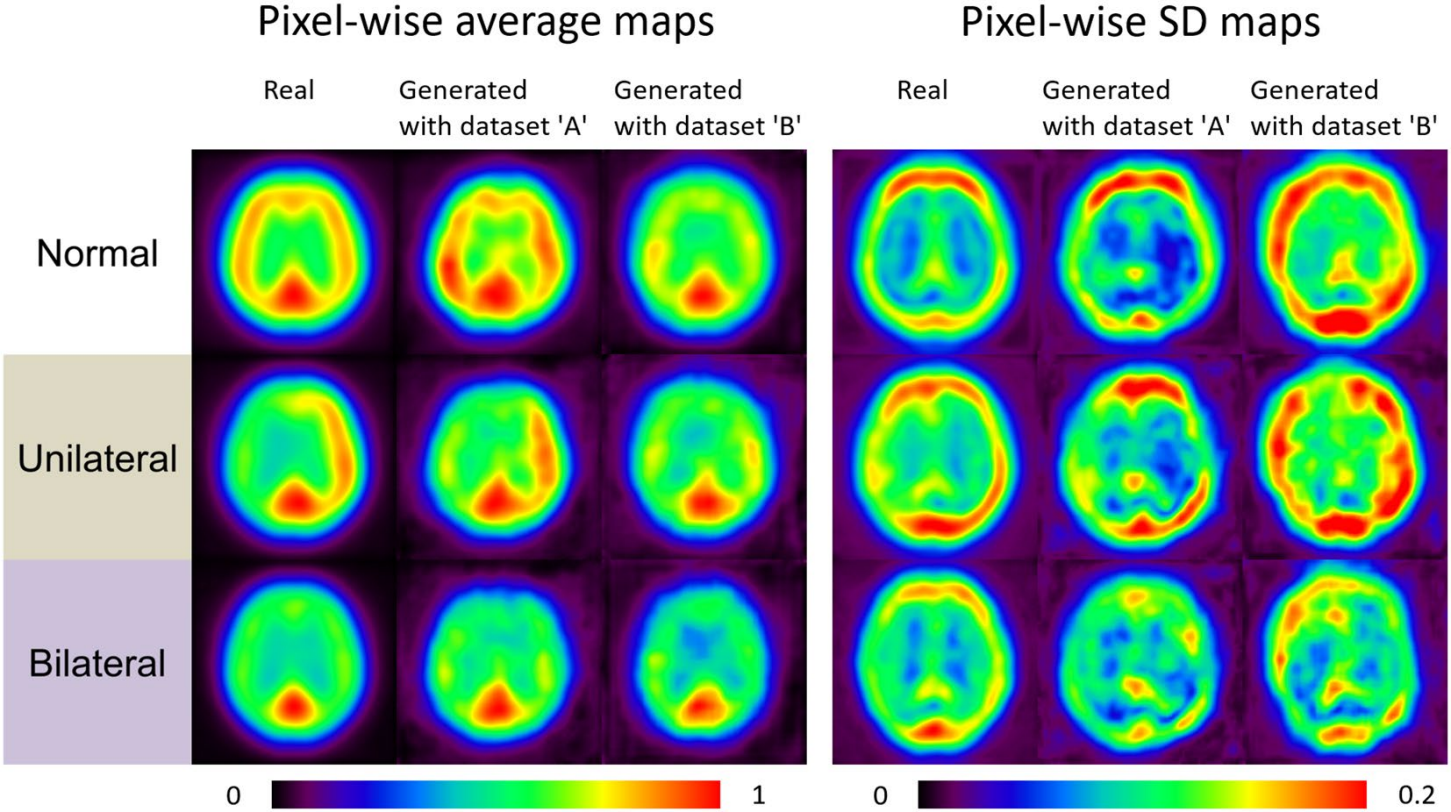
*Left:* pixel-wise average maps for real and generated images with dataset ‘A’ and ‘B’. *Right:* pixel-wise standard deviation (SD) maps for real and generated images with dataset ‘A’ and ‘B’.

For MC, ‘A’ revealed comparable findings when compared to real images, including normal (*P* = 0.8) and pathological scans (unilateral, *P* = 0.99; bilateral, *P* = 0.68). ‘B’ was significantly different for normal and bilateral defect patterns (*P* < 0.0001, respectively), but not for unilateral ischemic disease (*P* = 0.77). For LR, comparable results were recorded. For ‘A’, only uni- (*P* = 0.03), but not normal or bilateral defect scans (*P* ≥ 0.08) reached significance when compared to real images. For ‘B’, however, bilateral defects (*P* = 0.01), normal scans and unilateral ischemia (*P* < 0.0001, respectively) were significantly different (Fig. 5). As such, dataset ‘B’ was significantly different for virtually all investigated semi-quantitative parameters, while scans created by dataset ‘A’ were closely resembling to their real equivalents (except for unilateral disease on LR). Similar results were achieved for both pixel-wise average and SD. Both maps of normal and unilateral conditions for dataset ‘B’ were remarkably different from those calculated for dataset ‘A’ (Fig. 6).

## Discussion

Applying blood flow ^123^I-IMP SPECTs to our novel modified FastGAN, created scans were indistinguishable to acquired images from real patients, including normal studies and various degrees of ischemia. Although our developed neural network aimed at minimizing the number of training data, at least three anatomical compartments were still required to acquire images closely resembling scans of real patients. As such, if reasonable, but still rather limited amounts of supervised stimuli are provided, the applied FastGAN algorithm may allow to yield sufficient number of molecular brain scans for various clinical scenarios, e.g., for less balanced datasets in the context of orphan diseases or “data-hungry” deep learning technologies.

^123^I-IMP SPECTs have been frequently utilized to assess different degrees of cerebral ischemia, e.g., after head trauma^27^, stroke^28^, for identifying epileptogenic foci prior to surgical interventions or to differentiate between mild cognitive impairment and different types of dementia^29^. Of note, all of these studies enrolled a sufficiently large number of patients, while for other brain disorders, adequate patient recruitment may be challenging, e.g., to detect left or right hemispheric abnormalities in patients affected with Creutzfeldt-Jakob disease using ^123^I-IMP^30^. In this regard, augmenting cerebral blood flow scans of subjects with such an orphan disease may be helpful to test the clinical utility of this imaging modality, e.g., to differentiate between uni- or bilateral defect patterns. To the best of our knowledge, our modified FastGAN allowed for the first time to create artificial real equivalents using ^123^I-IMP SPECTs across a broad spectrum of disease patterns (Fig. 4). This technology is based on a neuronal network using both real images from actual patients fed to the GAN, a generator (trying to provide real images) and a discriminator (verifying whether the created scan is real or an imitation)^31^. The ongoing contest between both opponents along with a feedback loop will then help the discriminator to optimize its capability to determine which images should be classified as real, while the generator will learn creating scans more closely resembling real images^31^. As with every AI application, the number of initially provided scans serving as stimulus is of importance. As such, we aimed to reduce the number of needed real input images by applying only one skip-layer excitation, as such an approach allowed for minimizing the number of parameters needed to be learned along with a small matrix size of input images. Although dataset ‘A’ using CER, BG, and COR as input provided more realistic images than ‘B’ (only utilizing COR), we only applied a maximum of three anatomical compartments to create images that are indistinguishable to their real equivalents of patients (Fig. 5). Also partially explaining the superior performance of ‘A’ relative to ‘B’, the number of supervised data of normal, uni- and bilateral cerebral ischemia was rather imbalanced in the present study. For instance, representing diversity for each defect pattern, the radiotracer accumulation in pixel-wise SD maps of bilateral ischemia generated by dataset ‘A’ were lower than the real images, in particular for the frontal and occipital lobe (Fig. 6). This indicates that a lack of diversity within a specific pattern of cerebral ischemia may also lead to less realistic images. To overcome this issue, mini-batch standard deviation could be effective. In this regard, both the generator and discriminator develop consecutively, e.g., by adding more and more details during the training process, ultimately leading to further stabilization of the produced scans^32^. Taken together, the stimulus for the GAN should be carefully evaluated, e.g., for a bias of imbalanced supervised images for each defect pattern and anatomical location. Nonetheless, this may be challenging in a real-world scenario, where clinical cases provided to a GAN cannot always cover the entire spectrum of a certain disease^31^.

Moreover, as another limitation of this study, cerebral ischemia was restricted to three discrete patterns, ranging from normal scans, uni- and bilateral defects. Bigolin Lanfredi et al. have recently proposed a GAN model to visualize the progression of chronic obstructive pulmonary disease. The model incorporated a regression subnetwork to learn features in X-chest images for quantitative disease severity based on forced expiratory volume/forced lung capacity^33^ and such an adversarial regression training could also be incorporated on brain SPECTs. Another solution to reach the goal of quantitative severity in the generated images could be application of latent space. In this regard, a latent vector serving as input source of the desired image is searched. By editing that latent vector, variations of the desired image are generated. The use of latent space has been reported for natural images^34–36^, but it has also been used in the context of modality transformation for medical images^37^. As another limitation, our novel GAN was only applied to one specific disease using one single radiotracer and thus, our model should be validated among a broad spectrum of different radiopharmaceuticals for SPECT or positron emission tomography frequently applied in the clinic, e.g., ^18^F-labeled prostate-specific membrane antigen or somatostatin receptor directed PET^38–40^. Future studies should also address the aspect of three-dimensional images. A novel approach was recently proposed using ^123^I-ioflupane SPECT, which aimed to mimic characteristics of Parkinson's disease by integrating a transformer-based technique, which is based on a framework different from GAN^41^. In this regard, consecutive slices of a three-dimensional image were used and a neural network model was trained to generate slices for the rest of the region.

## Conclusion

We developed a light-weight GAN model for brain SPECT imaging that allowed us to create normal scans, but also varying degrees of cerebral ischemia closely resembling realistic images. In this context, we successfully applied a limited number of supervised data serving as input with a maximum of three anatomical compartments. As such, if reasonable amounts of supervised stimuli are provided, the applied FastGAN algorithm may allow to yield sufficient number of molecular brain scans for various clinical scenarios, e.g., for imbalanced datasets in the context of orphan diseases or “data-hungry” deep learning technologies.

## Data Availability

The datasets used and/or analysed during the current study are available from the corresponding author on reasonable request.
